# A Successful Method of Using Jaborandi in Acute and Chronic Bright’s Disease

**Published:** 1886-02

**Authors:** F. H. O’Brien

**Affiliations:** Atlanta, Ga.


### A SUCCESSFUL METHOD OF USING JABORANDI IN ACUTE AND CHRONIC BRIGHT'S DISEASE.

BY F. H. O'BRIEN, M. D., ATLANTA, GA.

REPORTED TO THE MEDICAL ASSOCIATION OF GEORGIA AT ITS THIRTY-SIXTH ANNUAL SESSION, SAVANNAH, APRIL 15, 1885.

Since the researches of Dr. Richard Bright, published in 1827, much has been said and written upon the subject, and with rapidly advancing knowledge of chemistry and microscopy, pathologists have done a great deal towards making our understanding of the condition of the kidneys and other organs in Bright's disease more clear. For our purpose, the kidney may be divided into three parts: first, tubes and malpighian bodies, which possess the gland elements; second, fibrous tissue, which binds the structures together; third, an abundant supply of blood vessels, which lie between the tubes, and some of the smallest size vessels enter into the composition of the malpighian bodies. Either of these parts may be the primary seat of disease, and the morbid change may be confined to the one structure or all may become diseased. In consequence of tubal disease or inflammation, the tubes become loaded with epithelium, or with fibrous matter, which exuded from the congested blood vessels, and the organ becomes enlarged and is called the large smooth kidney. This may present a pale or congested appearance. If seen early in the disease, it is congested, and is pale at a later stage. The frequency of recovery from such a condition would indicate that the other structures had entirely escaped, but if long continued, or the inflammatory action severe, an abnormal growth of fibrous tissue would ensue, and at last lead to granulation and contraction. In the early stage of granular kidney, the organ is enlarged, owing to the increased growth of fibrous tissue, and its characteristic appearance is that the surface, instead of being smooth, as in disease of the glandular structure, is studded with small, pale, round granulations, and the capsule is thickened and more firmly attached than in health ; whereas, in the first condition mentioned, the capsule is loose and of a healthy appearance. The increase of fibrous tissue is general throughout the entire organ, surrounding the malpighian bodies, and contraction takes place; the tubes are constricted, forming cysts, and such tubes as are not constricted are apt to undergo a morbid change in the later stages. The lardaceous or waxy kidney comes also under the head of Bright's disease, but differs from the varieties mentioned, from the fact that the changes which take place are not peculiar to kidney structure, but involve other organs and have their origin in the minute blood vessels. The glandular structure becomes infiltrated with a glassy material poured out by the small arteries; the organ becomes increased in size and firmness, has an anæmic look, and a cut surface presents the appearance of wax. It would be out of place, in a paper like this, for me to attempt to go into the many causes which might produce the conditions mentioned, or many symptoms concomitant, but there are some symptoms which the three varieties have in common, and I will speak of some of them in connection with remedial agents, and can say that the number of remedies extolled for their curative and palliative effects is large, and that many of them have failed to gain much favor in the professional mind. Jaborandi has, I think, gained more favor in the treatment of some of the symptoms than any other remedy. Farquharson says (page 358): "The powerful diaphoretic action of jaborandi, no less than its power in aiding the elimination of urea, would seem to suggest its use in various chronic kidney diseases (Bright's disease) as well as febrile condition. But its action is too short, sharp and sudden, and too much depression and inconvenience are produced to render us very hopeful of its ultimate success in practice, except in uremia, where pilocarpia may be given hypodermically." He also quotes Dr. Curschmann as saying: "Speaking from my own experience, I have found pilocarpia very useful in œdema, in dropsy of the cavities from heart or lung disease, and in chronic nephritis, . . . and that after diuretic, drastic and other means have failed. I believe a large field for its employment may be found in pleurisy, accompanied by serous exudation, both in promoting the absorption of this and preventing its re-accumulation after paracentesis. It is evidently indicated in chronic rheumatic affections, at least so far as these are amenable to diaphoretic treatment, and in some chronic skin affections its uses have been attended with considerable success."

Philips (Materia Medica and Therapeutics, page 139) says: "In 1875, M. M. Byasson and Hardy both obtained from the plant an alkaloid which is now known as pilocarpia. It is probable that jaborandi contains another alkaloid and a peculiar acid." Regarding the physiological effects, he says: "It produces two most marked effects, sweating and salivation, with a promptness and to a degree unequaled by any other known drug. The physiological effects resulting from continued use of the drug have not as yet been sufficiently studied, and it is by no means impossible that the violent perturbation of the system which results in the profuse sweating and salivation may impair the functional and even organic integrity of important organs; the sweating is apparently not due to increased afflux of blood to the surface, but rather to a paralysis of the inhibitory influence of that portion of the nervous system which presides over the sudatory apparatus."

Ringer says (7th edition): "I have used it in many cases of Bright's disease, not only without benefit, but, I must confess, not without doing harm, for it has always much depressed the patient and generally excited sickness, and effects have been so disagreeable that in almost every case the patient has begged the discontinuance of the treatment." Ringer concludes that, though of great physiological interest, jaborandi has not yet proved very serviceable in the treatment of disease.

Trousseau, in his "Treatise on Therapeutics" (Vol. 3), says: "We are much less certain of the therapeutic value of jaborandi than of its physiological properties. Therapeutical experiments, unfortunately, take much more time than physiological. We will state the results as at present known. The abundance of the secretions, and especially of the excretions, produced by jaborandi, led to its use as a derivative. This singular remedy has seemed most favorable in cases of humid asthma, that is, acute bronchitis contracted by the emphysematous, bronchitis accompanied by abundant catarrh, intense congestion, and sometimes even pulmonary œdema. Gubler quotes several cases of attacks of asthma which were relieved by the first administration of jaborandi. In influenza, as in acute bronchitis, the improvement has been equally rapid and decisive, but in bronchorrhœa the relief produced has been only transient. In acute dropsy, in acute pleurisy, jaborandi has seemed to be serviceable. The same has been the case in Bright's disease, acute articular rheumatism and muscular rheumatism. Jaborandi has produced palliation, but only for a time. Maillart, of Dijon, has been more successful in a case of articular gout retroceding to the lung, in which the action of jaborandi terminated the attack."

We see from the above-quoted authors that they all agree as to the certainty of the physiological action of jaborandi, and also that it is a powerful drug.

My conclusions, after having used it for several years, are: first, that it is a powerful remedy, the physiological effect of which is certain; second, that it is a remedy capable of doing great harm, if pushed to its full physiological effect or continued for any great length of time; third, that the primary effect is upon the skin, kidneys, salivary glands and heart, depressing the action of the latter organ. Having witnessed the good effect from the administration of a few doses, or I might say a single dose, of the remedy in acute kidney trouble, I felt hopeful of its success in chronic Bright's disease, if it could be taken by such patients for a considerable length of time, and to that end I have been directing my attention. The fact that two remedies combined, as for instance opium and belladonna, will produce the good effects of the drug desired without the bad effect following their administration when used alone has led me to combine with jaborandi several other remedies; but I find that the combination with it of nux vomica and digitalis answers the purpose better; and the three combined can be used for a considerable length of time with safety. I believe that jaborandi possesses powerful alterative properties when moderately given and the effect continued. I will relate my experience with it in a few well-marked cases, which I am satisfied no other remedy would have reached.

*Case 1.*—Woman, aged sixty (white.) Had been complaining eight or ten years with the numerous symptoms of Bright's disease. General appearance bad, anæmic, skin dusky, extremities œdematous. She complained of constant headache, neuralgic pains in different parts of the body, but more particularly about the joints of the lower extremities, dizziness, impairment of vision, loss of appetite, frequent attacks of vomiting and purging, after which she would feel better for a short time, dyspepsia, slight cough, drowsiness, urine scanty, skin dry and rough. On examination the urine was found to be highly colored, specific gravity 1032, with a small quantity of albumen, numerous casts, mostly of the granular variety, epithelial debris and a small amount of blood. Circulation feeble and rapid, and she frequently complained of palpitation. Her dropsical symptoms began about three years ago. She had been treated by several physicians without much benefit, and, as a last resort, she sought the advice and treatment of an irregular, who advertises to cure dropsy. She stated that he benefited her dropsy more than others had done, but, owing to her feeble condition, she could not stand the treatment, which consisted in being purged from eighteen to twenty-five times every other day.

I ordered for her one-third of a drachm of fluid extract jaborandi combined with nux vomica and digitalis, to be taken every third hour until the skin and kidneys were acting well, then to take three doses daily. At the end of three weeks she had taken four ounces of jaborandi, and upon examination no trace of œdema could be found. She said that it was the first time since the beginning of her dropsy (3 years) that it had entirely disappeared. Her general appearance was better, skin clearer, and she was gaining a little flesh. Her appetite improved; she slept well, feeling refreshed afterwards, and could take moderate exercise without fatigue. Examination of urine showed no albumen, specific gravity 1030, fewer casts and quantity larger than normal. The remedies were continued for two months, and cinchonia and iron given as a tonic. She has continued well since, with the exception of a few slight colds resulting from exposure, and whenever the functions of kidney or skin are interfered with, she takes a few doses of the mixture. She has been able to earn her own living for the past two years, with no return of her grave kidney symptoms.

*Case 2.*—Man; aged 48; a native of this State; business, wood-worker. Former diseases: Pneumonia, when he was twenty-two years of age, and a second attack when twenty-five; continued fever, twenty years ago (probably malarial). He had been healthy up to five years ago, when he contracted acute tubal nephritis, the result of attending a fire in midwinter, where he became saturated with water from the hose, remaining in this condition several hours, the water freezing to his clothing. He was confined to bed two weeks with dropsical and other symptoms. Improvement taking place; he returned to his business, but recovery was not complete, and as the degenerative changes went on, he complained of the numerous symptoms expected from chronic nephritis. After having been under the care of several physicians, he, too, sought the advice of an irregular, but his condition would not admit of the severe purging.

When he presented himself for treatment, the characteristic appearance of chronic Bright's disease was thoroughly marked. Extreme anæma and skin sallow. Physical examination revealed cardiac hypertrophy with mitral lesion, liver enormously enlarged, percussion showing that it extended nearly as high up as the nipple in front, and that the left lobe overlapped the stomach; he suffered considerable pain from that portion of the liver. The pleural cavities contained fluid, and also the peritoneal cavity. The circulation was bad, as the heart's action was irritable, rapid and intermittent; the radial artery had the whip-cord feel; temporal-arteries could be plainly seen. He complained mostly of dyspnœa, sometimes coming in paroxysms without being excited by exercise (uremic asthma), pain in back and region of kidneys, and in the epigastrium, increased by pressure, loss of appetite

(when food was taken it caused distress), occasional headache, and for a long time he had been troubled with spells of drowsiness, often being overcome by the feeling at his business or at the table. Urine scanty—the first specimen secured being about two ounces, all that had passed in the morning. It was loaded with casts, fibrous and epithelial, and a trace of albumen; specific gravity 1020. This case, I feared, might be too severe a test for jaborandi, as I thought the morbid changes were too extensive, and the envolvement of other organs too severe to allow much room for hope in the jaborandi treatment; but I determined to give it a fair trial, and the results were better than might have been expected. His improvement was rapid; the urine increased in quantity until a little more than a half-gallon was passed daily; the skin became active in its functions, thus enabling him to excrete rapidly through both channels; retained products; the circulation became better, and he was soon relieved of all of his most troublesome symptoms. The dropsy of the two cavities disappeared; his appetite returned; he slept well, and in less than three weeks he was able to walk from his house to my office and back, a distance of two miles.

After taking jaborandi for four or five weeks, he frequently remarked, "How poor I am getting!" It was particularly noticeable about his hands, legs and feet. They appeared shriveled, as if from extreme age, but within three months he had gained twenty-two pounds in weight. Six months afterwards he presented a healthy appearance; his color had returned, and when he walked in the cool air his hands, face and ears were tinged with a red blush, showing that the capillaries were capable of responding to the action of cold, which denoted a healthy action of the skin. His liver had been reduced in size to its normal proportion, except the left lobe, which was still enlarged, but not as much as at first. During his return to health he had several drawbacks. He returned to his business five weeks after treatment began (which was too soon), had a good deal of going up and down stairs to do, and unfortunately contracted a violent cold. His urine again became scanty, and a return to the mixture was ordered. It relieved him in a few days, and from time to time, as it was necessary, he has continued the mixture, and during the past year and a half has been able to attend to his business.

*Case 3.*—A car-builder; aged fifty-six; native of this country; former history good, except that he contracted syphilis twenty-two years ago and has had some manifestations of the trouble of late years. I saw him in consultation with two other physicians, and the diagnosis of amyloid kidney, due to syphilis, was made. He had been complaining for several months, but had only been confined to bed about a month. The condition when I saw him was extremely critical. He suffered greatly with dyspnœa, and had not been able to lie down for more than a week on account of effusion in the chest. He had general anasarca, and physical examination revealed enlargement of liver, spleen and heart; pulse intermittent. His urine contained one-third albumen and was very scant. The ordinary treatment for affording relief in such cases had been tried and had failed. He was put upon jaborandi treatment, and relief almost immediate was the result. In about two weeks he was entirely free from his dropsical trouble and expressed himself as feeling more comfortable than he had in months. Against the advice of his attending physician, he returned to his work, which was very hard, too soon, and relapsed into the same condition in about three weeks, this time placing himself under the care of a dropsy doctor, who promised to cure him in such a manner that his dropsy would not return for a year, if ever. After a severe purge he dropped dead while on his way to the water-closet.

*Case 4.*—Boy six years of age, with acute tubal nephritis, contracted two weeks after convalescence from continued fever. When my attention was directed to the case he had general anasarca. Temperature, 103; respiration, 40; pulse, 150, very feeble; chest filled with fluid and dyspnœa urgent. Consultation was requested and held and a very unfavorable prognosis made. He was placed upon the jaborandi treatment, which was continued for one week, when tonics were substituted, and the result was complete recovery. It has now been six months since his recovery, and the little fellow appears to be in perfect health.

I have frequently noticed that in using the jaborandi continuously, the specific gravity of the urine would remain the same, or even be increased as the secretion of urine increased, and also that casts would be more numerous in the beginning of the treatment. After decided improvement takes place the quantity of urine becomes less, until little more than the normal quantity is passed, and then as the quantity of urine decreases the specific gravity returns to normal.

I feel confident that jaborandi must act in some special manner upon the kidneys, assisting nature to free the tubes when they are obstructed with epithelial or fibrous debris. I would, of course, use other remedies in conjunction with the jaborandi mixture, if indicated, such as specific tonic, purgatives, etc.

I feel that jaborandi is entitled to the credit of possessing the power of bringing about, from its continued use, favorable results, and that Bright's disease is curable, in the same sense in which we apply the word cure to any other disease in which organic changes have taken place; or, in other words, a cure would consist in assisting nature to stop a morbid process, throwing off effete material and placing a barrier between diseased and healthy structure.

